# Prediction of Nonlinear Micro-Milling Force with a Novel Minimum Uncut Chip Thickness Model

**DOI:** 10.3390/mi12121495

**Published:** 2021-11-30

**Authors:** Tongshun Liu, Kedong Zhang, Gang Wang, Chengdong Wang

**Affiliations:** School of Mechanical and Electric Engineering, Soochow University, Suzhou 215021, China; tongshunliu@hotmail.com (T.L.); zhangkedong@suda.edu.cn (K.Z.); cdwang@suda.edu.cn (C.W.)

**Keywords:** micro-milling, minimum uncut chip thickness, cutting force, cutting force coefficient

## Abstract

The minimum uncut chip thickness (MUCT), dividing the cutting zone into the shear region and the ploughing region, has a strong nonlinear effect on the cutting force of micro-milling. Determining the MUCT value is fundamental in order to predict the micro-milling force. In this study, based on the assumption that the normal shear force and the normal ploughing force are equivalent at the MUCT point, a novel analytical MUCT model considering the comprehensive effect of shear stress, friction angle, ploughing coefficient and cutting-edge radius is constructed to determine the MUCT. Nonlinear piecewise cutting force coefficient functions with the novel MUCT as the break point are constructed to represent the distribution of the shear/ploughing force under the effect of the minimum uncut chip thickness. By integrating the cutting force coefficient function, the nonlinear micro-milling force is predicted. Theoretical analysis shows that the nonlinear cutting force coefficient function embedded with the novel MUCT is absolutely integrable, making the micro-milling force model more stable and accurate than the conventional models. Moreover, by considering different factors in the MUCT model, the proposed micro-milling force model is more flexible than the traditional models. Micro-milling experiments under different cutting conditions have verified the efficiency and improvement of the proposed micro-milling force model.

## 1. Introduction

Micro milling is viewed as a promising technology for manufacturing micro devices because of its high machining accuracy and the ability to cut a variety of materials [1]. The cutting force, which is related to many cutting performances such as the vibration [2], tool deflection [3], deflection of material [4,5], energy consumption [6,7] and machining quality [8], plays an important role in the micro-milling process [9]. Constructing an accurate cutting force model is of great significance to the application and development of micro-milling technology.

At the beginning of the 21st century, Bao and Tansel [10] first investigated the micro-milling force model. Over the past decade, different factors such as the tool runout [11], minimum uncut chip thickness (MUCT) [12], dead metal zone [13], chip thickness accumulation [14], tool wear [15] and elastic recovery [16] were included in the micro-milling force model, making the micro-milling force model more and more accurate. The MUCT, one of the most representative characteristics of the micro-cutting process, dividing the cutting zone into the shear and ploughing regions, has a significant effect on the cutting force of micro-milling. Determining the MUCT value is of great importance for predicting the micro-milling force.

Generally, the MUCT value could be obtained in three ways: Inferred from the measured cutting force and the surface profile, simulated by the finite element method and determined by an analytic MUCT model. Dib et al. [17] determined the minimum uncut chip thickness by means of cutting forces from the dynamometer. Skrzyniarz et al. [18] determined the minimum uncut chip thickness of micro turning from a surface profile obtained with a contact profilometer. Vogler et al. [19] found the minimum uncut chip thickness values in micro-milling ferrite and pearlite materials through finite element simulations. Due to the high flexibility and good interpretability, the analytic MUCT model is proven to be an efficient way to determine the MUCT value. Back in the 1970s, Abdelmoneim and Scrutton [20] constructed an analytical MUCT model for the precision machining process. With the rise of micro-machining since the 1990s, the analytical MUCT model has once again become a research hotspot. Liu et al. [21] deduced that the ratio of MUCT to the cutting-edge radius is a function of the effective flow stress and shear strength. By assuming that the shear angle is equal to the stagnant angle, Son et al. [22] expressed the stagnant angle corresponding to the MUCT as a function of the friction angle. Malekian et al. [23] concluded that the stagnant angle is equal to the friction angle and verified that the equation holds regardless of whether it is under the minimum cutting energy principle [24] or the infinite shear strain principle [25].

The studies above reveal that the MUCT value depends on many different factors such as the friction angle, shear strength and flow stress. However, each study only considered some of these factors. For a better understanding of the MUCT, a more comprehensive MUCT model is required. Moreover, the constraint condition, that the stagnant angle corresponding to the MUCT should be greater than the friction angle, was not taken into account in the existing MUCT models. As will be demonstrated in Section 2, the stagnant angle corresponding to the MUCT is a singularity of the shear/ploughing force distribution function. Without the constraint condition, the force distribution function is not integrable, leading to an infinite theoretical force and an unstable cutting force model. To obtain a finite theoretical cutting force and a stable cutting force model, the stagnant angle must be set greater than the friction angle in the MUCT model.

In this study, based on the assumption that the normal shear force and the normal ploughing force are equivalent at the MUCT point, a novel analytic MUCT model is constructed to determine the minimum uncut chip thickness. Nonlinear piecewise cutting force coefficient functions with the novel MUCT as the break point are constructed to represent the distribution of the shear/ploughing force under the effect of minimum uncut chip thickness. Under the proposed assumption, the stagnant angle corresponding to the MUCT is constantly greater than the friction angle, and the cutting force coefficient function is absolutely integrable, making the force prediction model more stable than the traditional models. Moreover, under the proposed equilibrium normal force assumption, different factors such as the friction angle, shear stress and ploughing coefficient are comprehensively included in the MUCT model, making the proposed cutting force model more flexible than the conventional models.

This paper evolves as follows. Section 2 introduces the related works and the prerequisite knowledge of this study. The novel comprehensive MUCT model is proposed in Section 3. In Section 4, the nonlinear micro-milling force model embedded with the novel MUCT is constructed. The model parameters are estimated in Section 5. The efficiency of the proposed model is validated by micro-milling experiments in Section 6.

## 2. Prerequisite Knowledge and Related Works

**Figure 1:**
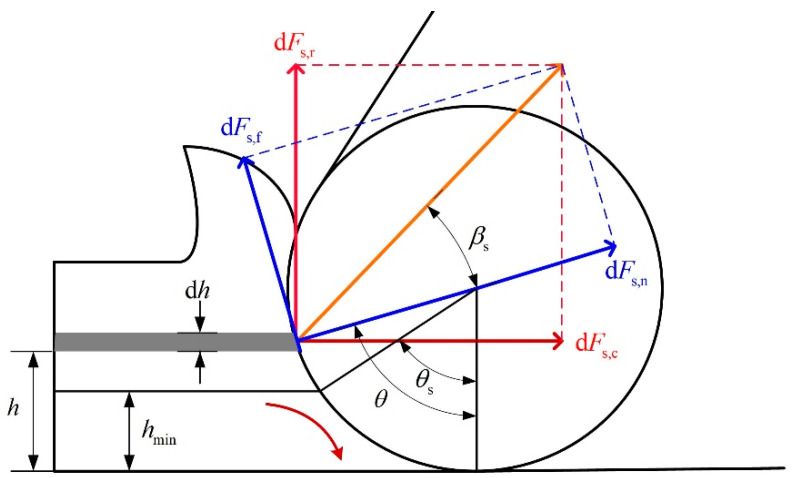
The distribution of cutting force under cutting edge radius size effect.

The comparability of the cutting-edge radius and the uncut chip thickness, viz. the cutting-edge radius size effect, leads to a minimum uncut phenomenon in micro-milling. As Figure 1 shows, under the cutting-edge radius size effect, the cutting region is divided into the shear region above the MUCT and the ploughing region under the MUCT.

There are two classical principles to model the cutting force in the shear region: The minimum cutting energy principle [24] and the maximum shear stress principle [26]. Under the minimum cutting energy principle, the distribution of the shear force on the round tool edge could be represented as:

(1) $$dF_{s,c}=\frac{\tau_{s}sin\left(\theta -\beta_{s}\right)}{{sin}^{2}\left(\frac{\theta -\beta_{s}}{2}\right)}dhdz$$

(2) $$dF_{s,r}=\frac{\tau_{s}cos\left(\theta -\beta_{s}\right)}{{sin}^{2}\left(\frac{\theta -\beta_{s}}{2}\right)}dhdz$$

where $dF_{s,c}$ is the tangential shear force, $dF_{s,r}$ is the radial shear force, dh is the unit uncut chip thickness (UCT) and dz is the unit axial cutting depth. $\tau_{s}$ is the shear stress and $\beta_{s}$ is the friction angle in the shear region. Notation θ is the angle corresponding to the UCT h, as shown in Figure 1. With the maximum shear stress principle, the distribution of the shear force on the round tool edge could be written as:

(3) $$dF_{s,c}=\frac{\sqrt{2}\tau_{s}sin\left(\theta -\beta_{s}\right)}{sin\left(\theta -\beta_{s}-\frac{\pi }{4}\right)}dhdz$$

(4) $$dF_{s,r}=\frac{\sqrt{2}\tau_{s}cos\left(\theta -\beta_{s}\right)}{sin\left(\theta -\beta_{s}-\frac{\pi }{4}\right)}dhdz$$

In the shear region with a negative rake angle ($\theta_{s}\leq \theta <\frac{\pi }{2}$), the chip flows towards the radial direction, and thus the radial shear force of the tool must be positive (as Figure 1 shows). To fulfill this condition, the stagnant angle should be set $\theta_{s}\geq \frac{\pi }{4}+\beta_{s}$ under the maximum shear stress principle shown in Equation (4). In the shear region on the round edge, the friction angle is relatively large. Therefore, with the maximum shear stress principle, the range of the shear region with a negative rake angle may be very narrow or even nonexistent. This is contradictory to the existing studies, which show that there exists a relatively broad shear region on the round edge with a negative rake angle. As Equation (2) shows, with the minimum energy principle, the radial shear force is constantly positive, and the potential range of the shear region on the round edge is broad enough. This makes the minimum energy principle more reasonable and flexible to model the shear process under the edge radius size effect. Therefore, the minimum cutting energy principle is adopted in this study.

Equations (1) and (2) imply that the friction angle is a singularity of the stress distribution function. The stress tends to be infinite when the angle θ approximates the friction angle. Moreover, if the shear region covers the friction angle (the stagnant angle is smaller than the friction angle), the stress distribution function is not integrable, and the total radial shear force $\int \;dF_{r}$ is infinite. This does not match the practical micro-milling process. Therefore, under the minimum cutting energy principle, the stagnant angle must be set greater than the friction angle in the MUCT model.

Although Son et al. [22] and Malekian et al. [23] have built friction-angle-included MUCT models, they do not consider the constraint condition wherein the stagnant angle should be greater than the friction angle. In this study, a novel MUCT model is derived by assuming that the normal shear force and the normal ploughing force are equivalent at the MUCT point. Under this assumption, the constraint condition that the stagnant angle should be greater than the friction angle is fulfilled. In addition, under the proposed assumption, the shear stress, friction angle and ploughing coefficient are comprehensively included in the MUCT model, making the proposed MUCT model more flexible to describe the edge radius size effect.

## 3. Novel Minimum Uncut Chip Thickness Model

The proposed assumption that the normal shear force and the normal ploughing force at the MUCT point are equivalent could be described by the following equation:

(5) $$\frac{\tau_{s}cos\left(\beta_{s}\right)}{{sin}^{2}\left(\frac{\theta_{s}-\beta_{s}}{2}\right)}dhdz=r_{e}\sigma_{m}d\theta dz$$

**Figure 2:**
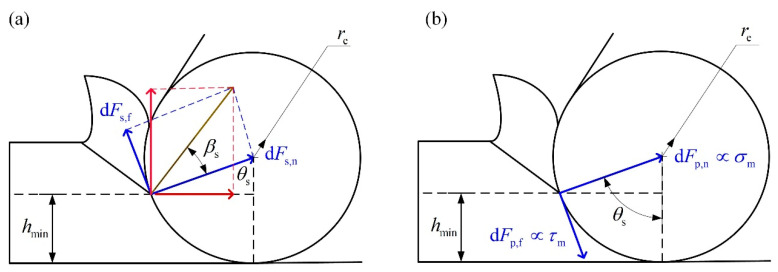
The distribution of the shear/ploughing force under the minimum uncut chip thickness. (**a**) The shear force above the minimum uncut chip thickness (MUCT) point; (**b**) the ploughing force under the MUCT point.

The left of Equation (5) is the normal shear force $dF_{s,n}$ above the MUCT point (Figure 2a), and the right is the normal ploughing force $dF_{p,n}$ under the MUCT point (Figure 2b). Notation $\;\tau_{s}$ is the shear stress. Notation $\beta_{s}$ is the friction angle in the shear region. Notation $\theta_{s}$ is the stagnant angle corresponding to the minimum uncut chip thickness $h_{\min}$. Notation $r_{e}$ is the cutting-edge radius. Notation $\sigma_{m}$ is the ploughing coefficient.

Taking $2\;{sin}^{2}\left(x\right)=1-cos\left(2x\right)$ and $dh=r_{e}sin\left(\theta_{s}\right)d\theta$ into Equation (5), there is:

(6) $$\frac{\sigma_{m}}{2\tau_{s}cos\beta_{s}}=\frac{sin\theta_{s}}{1-cos\left(\theta_{s}-\beta_{s}\right)}$$

Denoting $\frac{\sigma_{s}}{2\tau_{s}cos\beta_{s}}$ as k, Equation (6) could be rewritten as:

(7) $$sin\theta_{s}+kcos\left(\theta_{s}-\beta_{s}\right)=k$$

Decomposing $sin\theta_{s}$ into $sin\left(\theta_{s}-\beta_{s}\right)cos\left(\beta_{s}\right)+cos\left(\theta_{s}-\beta_{s}\right)sin\left(\beta_{s}\right)$ and dividing both sides of Equation (7) by $cos\beta_{s}$, Equation (7) could be written as:

(8) $$sin\left(\theta_{s}-\beta_{s}\right)+cos\left(\theta_{s}-\beta_{s}\right)\left(tan\beta_{s}+\frac{k}{cos\beta_{s}}\right)=\frac{k}{cos\beta_{s}}$$

Denoting $tan\beta_{s}+\frac{\sigma_{m}}{2\tau_{s}{cos}^{2}\beta_{s}}$ as tanγ, Equation (8) could be written as:

(9) $$sin\left(\theta_{s}-\beta_{s}+\gamma \right)=\frac{\sigma_{m}}{\sqrt{4\tau_{s}^{2}{cos}^{4}\beta_{s}+{\left(\tau_{s}sin2\beta_{s}+\sigma_{m}\right)}^{2}}}$$

where $\gamma =arctan\left\{tan\beta_{s}+\frac{\sigma_{m}}{2\tau_{s}{cos}^{2}\beta_{s}}\right\}$.

For Equation (9), there are two solutions near the friction angle, written as:

(10) $$\theta_{s1}=arcsin\left\{\frac{\sigma_{m}}{\sqrt{4\tau_{s}^{2}{cos}^{4}\beta_{s}+{\left(\tau_{s}sin2\beta_{s}+\sigma_{m}\right)}^{2}}}\right\}-arctan\left\{tan\beta_{s}+\frac{\sigma_{m}}{2\tau_{s}{cos}^{2}\beta_{s}}\right\}+\beta_{s}$$

(11) $$\theta_{s2}=\pi -arcsin\left\{\frac{\sigma_{m}}{\sqrt{4\tau_{s}^{2}{cos}^{4}\beta_{s}+{\left(\tau_{s}sin2\beta_{s}+\sigma_{m}\right)}^{2}}}\right\}-arctan\left\{tan\beta_{s}+\frac{\sigma_{m}}{2\tau_{s}{cos}^{2}\beta_{s}}\right\}+\beta_{s}$$

It could be proven that the solution $\theta_{s1}$ is smaller than the friction angle, and the solution $\theta_{s2}$ is constantly greater than the friction angle. To fulfill the constraint condition that the stagnant angle should be greater than the friction angle, the solution $\theta_{s2}$ is set as the theoretical stagnant angle $\theta_{s}=\theta_{s2}$. According to the geometric relationship shown in Figure 2, the MUCT corresponding to the stagnant angle $\theta_{s}$ is:

(12) $$h_{\min}=r_{e}\;\left(1-cos\theta_{s}\right)$$

According to Equations (11) and (12), it could be concluded that (1) the MUCT value is determined by the friction angle, the ratio of the ploughing coefficient $\sigma_{m}$ to the shear stress $\tau_{s}$ and the cutting edge radius $r_{e}$; (2) the MUCT linearly increases as the cutting edge radius increases; (3) the MUCT decreases as the ratio $\frac{\sigma_{m}}{\tau_{s}}$ increases; (4) if the ratio $\frac{\sigma_{m}}{\tau_{s}}$ tends to be infinite, the stagnant angle approximates to the friction angle, and the proposed MUCT model becomes the model in the study [23].

## 4. Micro-Milling Force Prediction with the Novel MUCT Model

The MUCT divides the cutting zone into shear and ploughing regions. If the uncut chip thickness is smaller than the MUCT, the micro-milling force results from the ploughing effect. Otherwise, the cutting force corresponds to the shear force. In this section, nonlinear cutting force coefficient functions embedded with the novel MUCT are constructed to represent the distribution of the shear/ploughing force under the effect of MUCT. The input of the nonlinear cutting force coefficient function is the uncut chip thickness, and the output of the nonlinear cutting force coefficient function is the distribution of the shear/ploughing force. The MUCT is a breakpoint of the nonlinear cutting force coefficient function and switches the shear/ploughing process.

**Figure 3:**
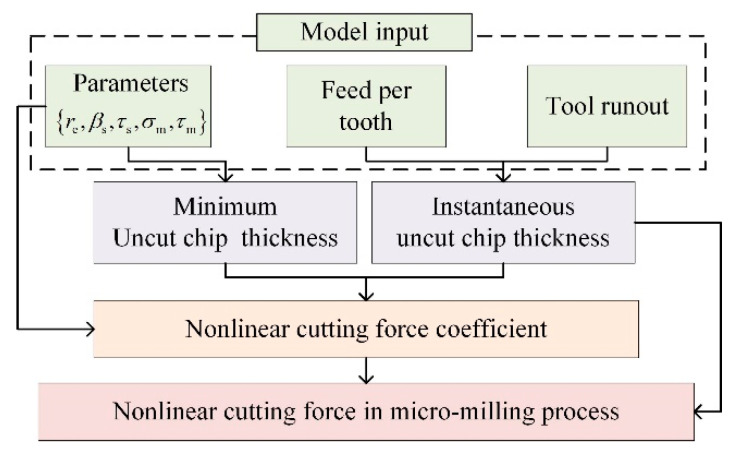
Model of the proposed micro-milling force embedded with the novel MUCT model.

The modeling flowchart is shown in Figure 3. Firstly, the uncut chip thickness model considering the tool runout is derived (Section 4.1). Then, nonlinear piecewise cutting force coefficient functions with the proposed MUCT as the breakpoint are constructed to reveal the nonlinear variation law of the shear/ploughing force with the uncut chip thickness (Section 4.2). Finally, the cutting force model is obtained by integrating the nonlinear cutting force coefficient functions (Section 4.3).

### 4.1. Uncut Chip Thickness Model

The equivalent cutting form in a previous study [15] is adopted to model the uncut chip thickness under tool runout. The UCT of the k−th equivalent radius at the cutting depth z could be written as:

(13) $$h_{k,z}\left(\phi \right)=max\left\{{min}_{m}\left[R_{k,z}-R_{m,z}+f_{z}sin\left(\phi_{k}^{z}\right)\frac{M\cdot \Delta \theta_{m,k}^{z}}{2\pi }\right],0\right\}$$

where $f_{z}$ is the feed per tooth, notation $R_{k,z}$ is the k−th equivalent radius at the cutting depth z, $\Delta \theta_{m,k}^{z}$ is the angle that the k−th equivalent radius clockwise leads the m−th equivalent radius to at the depth z. Notation ϕ is the reference position angle, $\phi_{k}^{z}$ is the rotation angle of the k−th equivalent radius at the depth z. The detailed process of determining the equivalent radius $R_{k,z}$ and the equivalent angles $\Delta \theta_{m,k}^{z}$ by the tool runout parameters can be found in reference [15].

### 4.2. Nonlinear Cutting Force Coefficient Function

**Figure 4:**
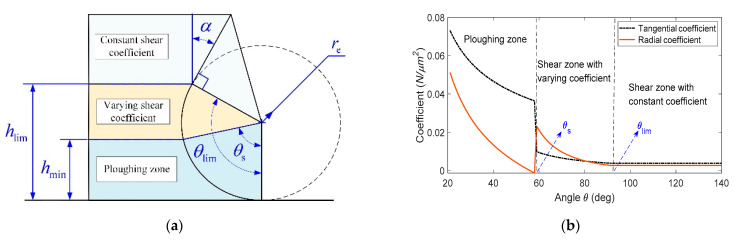
The nonlinear cutting force coefficient function. (**a**) The division of the cutting zone; (**b**) the nonlinear cutting force coefficient in three different cutting regions.

Nonlinear cutting force coefficient functions are constructed to represent the distribution of the shear/ploughing force on the cutting edge. For the convenience of mathematical representation, the uncut chip thickness h is transformed to the angle θ by transformation $\;\theta =\arccos\left(1-\frac{h}{r_{e}}\right)$ (Figure 4a). The ideal rake angel is α. The angle corresponding to the intersection point of the rake face and the round edge is $\theta_{\lim}$, determined by the equation $\theta_{\lim}=\alpha +\frac{\pi }{2}$. The uncut chip thickness corresponding to the angle $\theta_{\lim}$ is $h_{\lim}=r_{e}-r_{e}\;cos\theta_{\lim}$. The angle $\theta_{\lim}$ further divides the shear region into two parts: The part with ideal rake angle where the cutting coefficients do not vary with the uncut chip thickness, and the part of interval $\left[\theta_{s},\;\theta_{\lim}\right]$ in which the cutting force coefficients vary with the uncut chip thickness. Including the ploughing region, the whole cutting region has three parts with different cutting mechanisms (Figure 4b). According to the classical cutting theory and the ploughing mechanism, the distribution of the shear/ploughing force, viz. the cutting force coefficient function, could be written as:

(14) $$K_{c}\left(h|r_{e},\alpha ,\lambda \right)=\{\begin{array}{c} \frac{\tau_{s}sin\theta_{\lim}-\beta_{s}}{{sin}^{2}\left(\frac{\theta_{\lim}-\beta_{s}}{2}\right)}\;\;\;\;\;\;\;\;\;\;\;\;\;\;\;\;\;\;\;\;\;\;\;\;\;\;\;\;\;h\;\geq h_{\lim}\\ \frac{\tau_{s}sin\left(\theta -\beta_{s}\right)}{{sin}^{2}\left(\frac{\theta -\beta_{s}}{2}\right)}\;\;\;\;\;\;\;\;\;\;\;\;\;\;\;\;\;\;\;h_{\min}\;\leq h<h_{\lim}\\ \sigma_{m}+\tau_{m}cot\theta \;\;\;\;\;\;\;\;\;\;\;\;\;\;\;\;\;\;\;\;\;\;\;\;0\leq h\leq h_{\min} \end{array}$$

(15) $$K_{r}\left(h|r_{e},\alpha ,\lambda \right)=\{\begin{array}{c} \frac{\tau_{s}cos\theta_{\lim}-\beta_{s}}{{sin}^{2}\left(\frac{\theta_{\lim}-\beta_{s}}{2}\right)}\;\;\;\;\;\;\;\;\;\;\;\;\;\;\;\;\;\;\;\;\;\;\;\;\;\;\;\;\;h\;\geq h_{\lim}\\ \frac{\tau_{s}cos(\theta -\beta_{s})}{{sin}^{2}\left(\frac{\theta -\beta_{s}}{2}\right)}\;\;\;\;\;\;\;\;\;\;\;\;\;\;\;\;\;\;\;h_{\min}\;\leq h<h_{\lim}\\ \sigma_{m}cot\theta -\tau_{m}\;\;\;\;\;\;\;\;\;\;\;\;\;\;\;\;\;\;\;\;\;\;\;\;0\leq h\leq h_{\min} \end{array}$$

Notation $K_{c}$ is the tangential cutting force coefficient function, and $K_{r}$ is the radial cutting force coefficient function. Notation h is the uncut chip thickness in Equation (13). The mechanical parameters set λ include the shear stress $\tau_{s}$, friction angle $\beta_{s}$ in the shear region, the ploughing coefficient $\sigma_{m}$ and the friction stress $\tau_{m}$ in the ploughing region. The MUCT $\;h_{\min}$ in Equations (14) and (15) are determined by the analytical MUCT model proposed in Section 2.

The distribution form of the cutting force in Equations (14) and (15) is valid for a ductile material. Due to the brittle–ductile transition phenomenon in cutting brittle material [27], the cutting force of brittle material is quite different from Equations (14) and (15). Therefore, the proposed cutting force model cannot be used to predict the cutting force when micro-milling a brittle material. In addition, the distribution form of the cutting force in Equations (14) and (15) is derived under an orthogonal cutting assumption. For a better understanding of the three-dimensional (3-D) micro-milling force, the oblique cutting assumption should be adopted, and the helix angle should be considered in Equations (14) and (15). In this study, to reduce the calculation cost, the micro-milling force is predicted by dividing the cutting part into thin disks along the axial direction, and the cutting process of each disk is approximated, regarded as orthogonal cutting.

### 4.3. Micro-Milling Force Model Embed with the Novel MUCT

The mechanical micro-milling force model embedded in the novel MUCT is mathematically represented as:

(16) $$dF_{c}=K_{c}\left(h|r_{e},\alpha ,\lambda \right)\cdot dh\cdot dz$$

(17) $$dF_{r}=K_{r}\left(h|r_{e},\alpha ,\lambda \right)\cdot dh\cdot dz$$

Notation dh is the differential of the uncut chip thickness, dz is the differential of the axial cutting depth. Notation $dF_{c}$ is the partial tangential force, while $dF_{r}$ is the partial radial force. By decomposing the partial forces into X and Y directions and integrating the partial forces, the theoretical cutting forces in the X-direction and the Y-direction could be represented as:

(18) $$F_{x}\left(\phi \right)=\overset{M}{\underset{k=1}{\sum }}\int_{z=0}^{d}\int_{h=0}^{h_{k,z}\left(\phi \right)}\left[K_{c}\left(h|r_{e},\alpha ,\lambda \right)\cdot cos\left(\phi_{k}^{z}\right)+K_{r}\left(h|r_{e},\alpha ,\lambda \right)\cdot sin\left(\phi_{k}^{z}\right)\right]\cdot dh\cdot dz$$

(19) $$F_{y}\left(\phi \right)=\overset{M}{\underset{k=1}{\sum }}\int_{z=0}^{d}\int_{h=0}^{h_{k,z}\left(\phi \right)}\left[K_{c}\left(h|r_{e},\alpha ,\lambda \right)\cdot sin\left(\phi_{k}^{z}\right)-K_{r}\left(h|r_{e},\alpha ,\lambda \right)\cdot cos\left(\phi_{k}^{z}\right)\right]\cdot dh\cdot dz$$

where notation d is the axial cutting depth. As the stagnant angle corresponding to the MUCT is constantly greater than the friction angle, the cutting force coefficient functions defined in Equations (14) and (15) are absolutely integrable. This means the theoretical cutting force, viz. the integral of the cutting force coefficient function, is limited, and the numerical integration calculation process of Equations (18) and (19) is stable. Without the constraint condition wherein the stagnant angle should be greater than the friction angle, the conventional nonlinear cutting force coefficient function is not integrable, and the stability of the numerical integration cannot be guaranteed.

## 5. Parameters Estimation

The forces in X and Y directions defined in Equations (18) and (19) are adopted to estimate the model parameters. Table 1 lists the two kinds of parameters to be estimated. The first one is the mechanical parameter consisting of the shear stress, friction angle, ploughing coefficient and friction stress in the ploughing region. The second one constitutes the parameters related to the tool moving trajectory, including the tool runout length, tool runout angle and the sampling point ϕ corresponding to the starting reference position angle $0^{\circ }$. The seven parameters are denoted as $\zeta =\left\{\phi ,r_{o},\gamma_{o},\beta_{s},\sigma_{m},\tau_{m},\tau_{s}\right\}$. The theoretical forces in X and Y directions in a shot cutting pass are denoted by the vectors $F_{x}$ and $F_{y}$. The measured forces in X and Y directions are denoted by the vectors ${\hat{F}}_{x}$ and ${\hat{F}}_{y}$. The purpose of the parameter estimation is to determine the optimum parameters, such that the discrepancy between the measured forces and the theoretical forces is smallest. Because there are many parameters to be estimated, the optimization process is prone to falling into a locally optimal solution. The genetic algorithm can find the global optimal solution and overcome the local optimal solution problem by imitating the natural selection and genetic mechanism. Therefore, the genetic optimization algorithm is adopted to estimate the model parameters in this study. The genetic optimization-based estimation process is expressed as:

(20) $$\min\left\{\|{\hat{F}}_{x}-F_{x}{\left(\zeta \right)\|}_{2}+\|{\hat{F}}_{y}-F_{y}{\left(\zeta \right)\|}_{2}\right\}\;\zeta =\left\{\phi ,r_{o},\gamma_{o},\beta_{s},\sigma_{m},\tau_{m},\tau_{s}\right\}\in \Omega \;s.t.\;h_{\min}\left(\beta_{s},\sigma_{m},\tau_{m},\tau_{s},r_{e}\right)<0.5r_{e}$$

The range of the parameters Ω is determined by the lower bound and the upper bound of the parameters. The detailed setting of the two bounds is presented in the experimental validation section. The inequality constraint $\;h_{\min}<0.5r_{e}$ is added to ensure the range of the shear region with a negative rake angle is broad enough. Since the proposed stagnant angle $\theta_{s}$ in Equation (11) is always greater than the friction angle $\beta_{s}$, the inequality constraint $\beta_{s}<\theta_{s}\left(\beta_{s},\sigma_{m},\tau_{m}\right)$ is not considered in the optimization-based estimation process.

## 6. Experimental Validation

### 6.1. Experimental Setup

**Figure 5:**
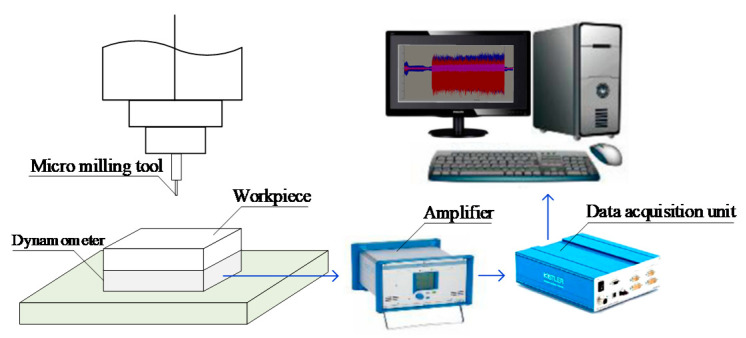
Experimental setup.

**Figure 6:**
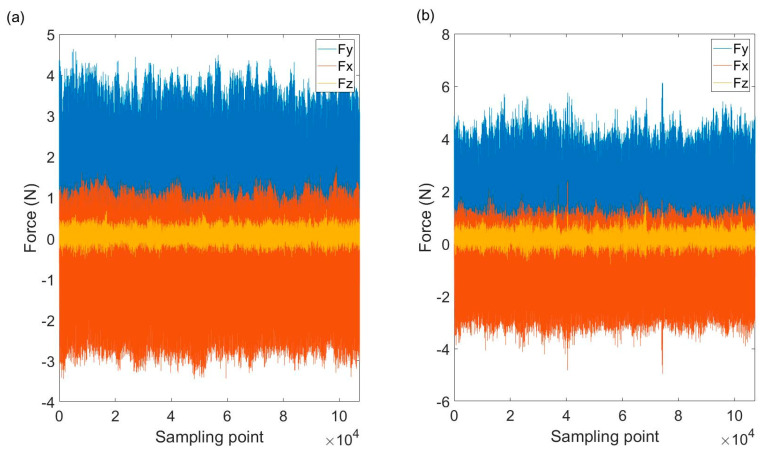
The profile of the measured cutting force. (**a**) The measured cutting force of C1; (**b**) the measured cutting force of C2.

Micro slot milling experiments are conducted to estimate the model parameters and verify the proposed micro-milling force model. A total of nine experiments are conducted with different spindle speeds, axial cutting depths and feeds per tooth. The cutting conditions of the nine experiments are listed in Table 2. In each experiment, two 3-cm-long slots are machined. The cutting force generated in the first slot is used to estimate the model parameters, and the cutting force of the second slot is adopted to verify the prediction accuracy of the model. The machine used in the experiments is the MIKRON HSM600U (Mikron Group, Agno, Switzerland) vertical milling machine. The tool is CS2008-0200 (UNION TOOL CO., Tokyo, Japan), whose diameter is 0.8 mm with two flutes. The helix angle of the tool is 30°. The cutting-edge radius is 2 μm. Steel AISI4340 is used as the work-piece material. The cutting force in three orthogonal directions is measured with a Kistler9119AA2 3-channel dynamometer (Kistler Precision Machinery (Shanghai) Co., LTD, Shanghai, China). The sampling rate is adaptively set according to the spindle rotation speed, such that the number of the sampling point in one spindle rotation cycle is consistent for the experiments with different rotation speeds. In this study, the number of the sampling point in one spindle rotation cycle is set to 180. The experimental setup is shown in Figure 5. The profile of the measured cutting force is shown in Figure 6.

### 6.2. Parameters Estimation Results

The seven parameters listed in Table 3 are estimated via the genetic optimization algorithm. The lower bound of the parameter set $\left\{\phi ,r_{o},\gamma_{o},\beta_{s},\sigma_{m},\tau_{m},\tau_{s}\right\}$ in Table 3 is [1, 0, 0, 0, 0, 0, 0] and the upper bound is $\left[180,3,\pi ,\frac{\pi }{4},1,1,5\times 10^{-3}\right]$. Table 3 shows that the calculated results are between the predefined lower bound and the upper bound. Therefore, the calibration result is valid.

Table 3 shows that the average shear stress $\tau_{s}$ under a cutting speed of 18,000 rpm (C1, C2, C3) is 0.99 Gpa, while the average shear stress $\tau_{s}$ under a cutting speed of 24,000 rpm (C4, C6, C7) is 1.01 Gpa and the average value under a cutting speed of 36,000 rpm (C5, C8, C9) is 1.05 Gpa. This implies the shear stress increases as the cutting speed increases. As shown in Table 3, the average ploughing coefficient $\sigma_{m}$ under a cutting speed of 18,000 rpm (C1, C2, C3) is 2.5 Gpa, while the average value under a cutting speed of 24,000 rpm (C4, C6, C7) is 2.6 Gpa and the average value under a cutting speed of 36,000 rpm (C5, C8, C9) is 2.9 Gpa. Therefore, it could be concluded that the ploughing coefficient $\sigma_{m}$ also increases as the cutting speed increasers. The ploughing coefficient $\sigma_{m}$ represents the extrusion stress of the tool to the workpiece. Micro-milling is an intermittent cutting process, and the extrusion process can be regarded as the impact of the tool on the workpiece. In this sense, the ploughing coefficient $\sigma_{m}$ reflects the impact force of the tool on the workpiece. Because the impact force increases with increasing speed, the ploughing coefficient $\sigma_{m}$ increases as the cutting speed increases.

The calibrated MUCT value and the corresponding stagnant angle are listed in Table 4. It could be found that the stagnant angle is greater than the friction angle listed in Table 3. This is consistent with the analysis in Section 2. The ratio of the MUCT value to the edge radius is also presented in Table 4. It clearly shows that the ratio of the MUCT value to the edge radius is in the range of (0.25–0.36). This is consistent with the findings in most of the previous research, which indicate the ratio is around 0.3 [28,29].

### 6.3. Force Prediction Results

**Figure 7:**
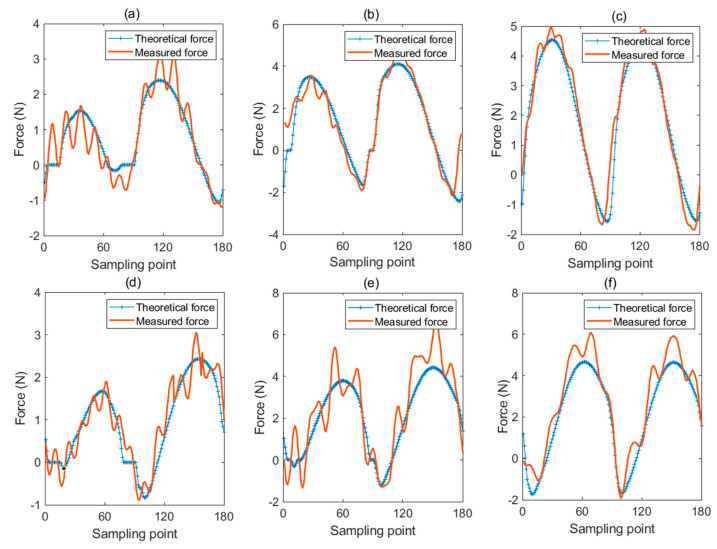
The predicted micro-milling force. (**a**) X-direction force of C1; (**b**) X-direction force of C2; (**c**) X-direction force of C3; (**d**) Y-direction force of C1; (**e**) Y-direction force of C2; (**f**) Y-direction force of C3.

The micro-milling force is predicted via the proposed force model embedded with the novel MUCT model. The relative error listed in the fifth column of Table 5 is adopted to examine the effectiveness of the model. The average prediction error for experiments with $f_{z}=2\;\mu m$ (C1, C7, C8) is 22.90%, while the average prediction error for experiments with $f_{z}=4\;\mu m$ (C2, C6, C9) is 21.76% and the average prediction error for experiments with $f_{z}=6\;\mu m$ (C3, C4, C5) is 15.57%. This implies that the prediction error increases as the feed per tooth decreases. The small feed per tooth increases the ploughing time in one spindle rotation cycle, and enhances the elastic recovery effect and vibration, leading to a drastically fluctuating force and an increasing force prediction error. This conclusion could also be verified by Figure 7 where the predicted cutting forces in experiments with different feed per tooth (C1–C3) are presented. The starting position angle corresponding to the first sampling point of the predicted force in Figure 7 is $0^{\circ }$. As discussed in Section 5, the parameter ϕ is the sampling point of the measured force corresponding to the starting position angle $0^{\circ }$. By optimizing the parameter ϕ via the genetic optimization algorithm in Equation (20), the measured cutting force can be aligned with the predicted cutting force. The optimum ϕ is listed in the second column of Table 3. As Figure 7 shows, with the optimum ϕ, the measured cutting force is well synchronized with the predicted cutting force.

### 6.4. Discussion on the Advantages of the Model

The MUCT models proposed by Son et al. [22] and Malekian et al. [23] are often utilized to predict the nonlinear micro-milling force. In this section, the model proposed in this paper is compared to Malekian’s model and Son’s model.

**Figure 8:**
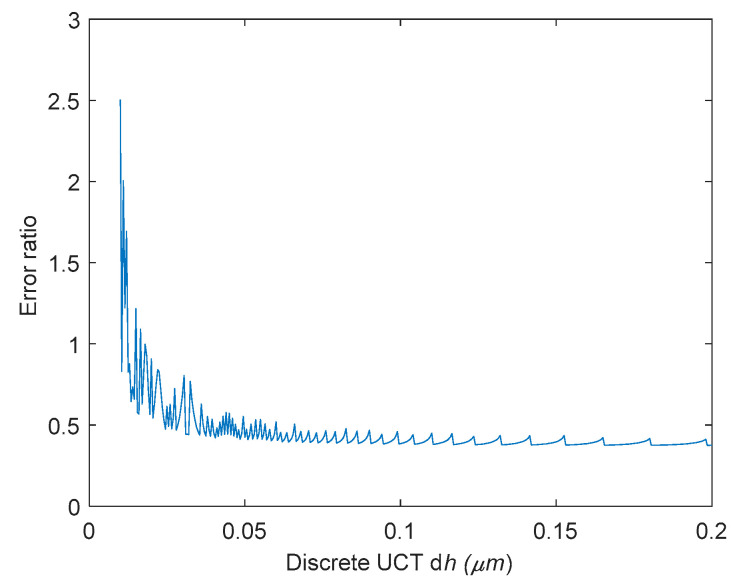
The prediction error vs. the discrete uncut chip thickness (UCT) with the approach of partial rake angle.

In Malekian’s MUCT model, the stagnant angle equals to the friction angle. As discussed in Section 2, the stress distribution function (cutting force coefficient function) is not integrable and the theoretical force is infinite if Malekian’s MUCT model is adopted. This problem could be addressed by two approaches: (1) Utilizing the average rake angle to represent the UCT-varying effective rake angle [30]; (2) the partial rake angle with discrete UCT [31,32]. Because the stress distribution function is a strong nonlinear function of the effective rake angle, the average rake angle and the simplified average stress cannot represent the nonlinear stress distribution. Therefore, the average rake angle approach may have a high prediction error. This could be verified by the prediction results presented in the second column of Table 5. For the approach of the partial rake angle with discrete UCT, it is difficult to select a proper discrete UCT d*h*. Figure 8 shows that the prediction error varies with d*h*, and the stability of the numerical calculation with the discrete UCT approach is poor. In this study, to find the optimal discrete UCT d*h* for the partial rake angle approach, the d*h* is set as a parameter to be estimated. According to the genetic optimization algorithm, the optimal d*h* for each experiment is estimated. Then, the partial rake angle approach with the optimal discrete UCT is adopted to predict the micro-milling force. The prediction error listed in the third column of Table 5 shows the force prediction error of the partial rake angle approach with the optimal discrete UCT is still much higher than the approach proposed in this study.

Under the minimum cutting energy principle and the assumption that the shear angle is equal to the stagnation angle, Son et al. [22] derived the stagnant angle as a linear function of the friction angle. The linear relationship between the stagnant angle and the friction angle in Son’s model could be represented by the equation $\theta_{s}=\frac{\pi }{4}-\frac{\beta_{s}}{2}$. To fulfill the constraint condition wherein the stagnant angle should be greater than the friction angle, the friction angle should be restricted in the range of $\beta_{s}\in \left(0,\frac{\pi }{6}\right)$ if Son’s model is adopted. The micro-milling force is also predicted by the cutting force model embedded with Son’s MUCT under the constraint condition $\beta_{s}\in \left(0,\frac{\pi }{6}\right)$. The force prediction error is listed in the third column of Table 5. As the constraint condition is considered, the stability of the force prediction process could be guaranteed, and the prediction accuracy is improved compared to Malekian’s model. However, as Son’s MUCT model only considers the friction angle, the flexibility of the model is lower than the proposed model in this study, and the prediction error is still higher than the proposed model.

The comparison above clearly shows that the proposed micro-milling force model embedded with the novel MUCT model is more flexible and more stable than the conventional models. The better flexibility and stability cause the cutting force prediction accuracy of the proposed model to be higher than the conventional models. As Table 5 shows, the prediction accuracy of the proposed model is improved by 5%–10% compared to the traditional models.

It should be noticed that all of the MUCT models mentioned above are derived under the minimum cutting energy principle. As discussed in Section 2, compared to the maximum shear stress principle, the minimum cutting energy principle is more reasonable to model the cutting force in the shear region on the round edge. In this section, the MUCT models with the maximum shear stress principle are also adopted to predict the micro-milling force. The average errors for different MUCT models with the maximum shear stress principle are as follows: 35.12% for Malekian’s model, 33.79% for Son’s model and 26.48% for our model. It clearly shows that the prediction error with the minimum cutting energy principle listed in Table 5 is much lower than the error under the maximum shear stress principle. This result further implies that the minimum cutting energy principle is more reasonable to model the cutting force on the round edge.

## 7. Conclusions

Based on the minimum cutting energy principle and the proposed equilibrium normal force assumption, a novel MUCT model considering the comprehensive effect of the shear stress, friction angle, ploughing coefficient and cutting-edge radius is constructed to determine the minimum uncut chip thickness in micro-milling. A nonlinear cutting force coefficient function embedded with the novel MUCT is constructed to represent the distribution of shear/ploughing force under the effect of minimum uncut chip thickness. By integrating the proposed cutting force coefficient functions, the nonlinear micro-milling force is accurately predicted. The conclusions are as follows:

- Compared to the maximum shear stress principle, the minimum cutting energy principle is more reasonable to model the cutting force in the shear region on the round edge.
- Under the proposed equilibrium normal force assumption, the stagnant angle corresponding to the MUCT is constantly greater than the friction angle, resulting in an integrable stress distribution function and a stable cutting force model.
- Embedded with a more flexible and stable MUCT model, the proposed nonlinear micro-milling force model is more accurate than the conventional models. The prediction accuracy of the proposed model is improved by 5%–10% compared to the traditional models.

The main contribution of this paper is proposing a novel analytical MUCT model with high flexibility and stability for predicting the micro-milling force. As more factors are considered, the proposed MUCT model is more flexible than the traditional models. Embedded with the novel MUCT, the distribution function of the shear/ploughing force is absolutely integrable and the micro-milling force model is numerically stable. In addition to the prediction of cutting force, the proposed MUCT model and the micro-milling force model could be further adopted to predict the tool deflection, vibration and energy consumption in the micro-milling process.

## Figures and Tables

**Table 1:** Parameters to be estimated.

The Type of Parameters	Parameters	Notation	Unit
mechanical parameters	shear stress	$\tau_{s}$	Gpa
	friction angle	$\beta_{s}$	rad
	ploughing coefficient	$\sigma_{m}$	Gpa
	friction stress in ploughing region	$\tau_{m}$	Gpa
parameters related to cutting trajectory	runout length	$r_{o}$	μm
	runout angle	$\gamma_{o}$	rad
	starting point.	ϕ	--

**Table 2:** Cutting conditions.

Cutting Condition	Spindle Speed (rpm)	Cutting SPEED (m/min)	Axial Cutting Depth (μm)	Feed Speed (mm/min)	Feed Speed per Tooth (μm/tooth)
C1	18,000	45.24	60	72	2
C2	18,000	45.24	80	144	4
C3	18,000	45.24	100	216	6
C4	24,000	60.32	80	288	6
C5	30,000	75.40	60	360	6
C6	24,000	60.32	60	192	4
C7	24,000	60.32	100	96	2
C8	30,000	75.40	80	120	2
C9	30,000	75.40	100	240	4

**Table 3:** Parameter calibration results.

Cutting Condition	ϕ	$r_{o}$	$\gamma_{o}$	$\beta_{s}$	$\sigma_{m}$	$\tau_{m}$	$\tau_{s}$
C1	30	1.01	2.07	0.52 (29.91°)	25.00	16.00	0.98
C2	29	1.02	2.05	0.56 (31.91°)	27.00	26.00	1.02
C3	35	0.09	1.43	0.53 (30.19°)	23.00	11.00	0.98
C4	124	0.92	0.86	0.50 (28.71°)	23.00	11.00	0.95
C5	22	0.76	2.16	0.51 (29.45°)	24.00	17.00	1.02
C6	38	1.17	2.68	0.57 (32.77°)	32.00	21.00	1.04
C7	123	0.32	0.95	0.44 (25.25°)	24.00	12.00	1.04
C8	29	0.21	1.69	0.45 (24.98°)	29.00	14.00	1.05
C9	23	0.36	1.08	0.60 (34.38°)	35.00	19.00	1.07

**Table 4:** Calibrated MUCT value.

	C1	C2	C3	C4	C5	C6	C7	C8	C9
$\theta_{s}$	48.45°	49.74°	49.68°	48.62°	48.42°	48.96°	43.74°	41.53°	49.80°
$h_{\min}$	0.673	0.7073	0.7061	0.6780	0.6725	0.6868	0.5550	0.5029	0.7092
$\frac{h_{\min}}{r_{e}}$	0.3367	0.3537	0.3530	0.3390	0.3363	0.3434	0.2775	0.2514	0.3546

**Table 5:** The cutting force prediction error of different models.

Cutting Condition	Malekian’s Model (Average Rake Angle)	Malekian’s Model (Partial Rake Angle)	Son’s Model under $\beta_{s}\in \left(0,\frac{\pi }{6}\right)$	Our Model
C1	36.19%	35.46%	29.36%	26.90%
C2	34.64%	33.55%	26.95%	23.29%
C3	20.78%	21.05%	17.92%	10.16%
C4	24.32%	26.79%	22.45%	15.38%
C5	33.31%	30.95%	24.53%	21.18%
C6	30.09%	29.30%	26.64%	21.17%
C7	26.32%	27.15%	23.77%	18.04%
C8	32.50%	33.76%	26.40%	22.14%
C9	32.88%	31.24%	25.23%	20.81%
Average of C1–C9	30.11%	29.92%	24.81%	19.10%

## Nomenclature

$h_{\min}$	Minimum uncut chip thickness (μm)
$\theta_{s}$	Stagnant angle (rad)
$\beta_{s}$	Friction angle (rad)
$\sigma_{m}$	Ploughing coefficient (Gpa)
$\tau_{m}$	Friction stress in ploughing region (Gpa)
$\tau_{s}$	Shear stress (Gpa)
$r_{e}$	Cutting edge radius (μm)
$r_{o}$	Length of tool runout (μm)
$\gamma_{o}$	Angle of tool runout (rad)
$K_{c}$	Shear-ploughing coefficient function in tangential direction (Gpa)
$K_{r}$	Shear-ploughing coefficient function in radial direction (Gpa)
